# Efficacy and safety of venetoclax in patients with relapsed/refractory multiple myeloma: a meta-analysis

**DOI:** 10.1186/s12885-023-11553-3

**Published:** 2023-11-03

**Authors:** Xiaohui Gao, Hui Zeng, Xiaoyan Zhao, Haibing Wu, Minchao Yan, Yuan Li, Gang Zhang, Fei Sun

**Affiliations:** 1 Departments of Pediatrics, The Affiliated Hospital of Jiaxing University, Jiaxing, 314000 Zhejiang China; 2 Departments of Hematology, The Affiliated Hospital of Jiaxing University, Jiaxing, 314000 Zhejiang China

**Keywords:** Venetoclax, Multiple Myeloma, Efficacy, Adverse events, Meta-analysis

## Abstract

**Background:**

Venetoclax is clinically active in treating relapsed/refractory multiple myeloma (RRMM). This study evaluated the efficacy and safety of venetoclax or venetoclax with other agents in treating RRMM.

**Methods:**

PubMed, Web of Science, Embase, and Cochrane Library were comprehensively searched. We included studies investigating the efficacy and safety of venetoclax or venetoclax with other agents in treating RRMM. Overall response rates (ORR), stringent complete response rates (sCR), complete response rates (CR), very good partial response rates (VGPR), partial response rates (PR), stable disease (SD), progressive disease (PD) and adverse events were synthesized using either a random-effects model or a fixed-effects model.

**Results:**

A total of 7 clinical trials with 482 patients with RRMM were included. Concerning venetoclax with other agents, the pooled ORR, sCR, CR, VGPR, PR, SD, and PD were 0.76 (95% CIs: 0.62, 0.87), 0.11 (95% CIs: 0.04, 0.21), 0.18 (95% CIs: 0.11, 0.26), 0.16 (95% CIs: 0.12, 0.25), 0.29 (95% CIs: 0.25, 0.34), 0.07 (95% CIs: 0.05, 0.10), and 0.11 (95% CIs: 0.04, 0.23). The overall rate of adverse events ≥ Grade 3 was 0.84 (95% CIs: 0.77, 0.91). The most common non-hematologic adverse events were nausea, diarrhea, fatigue, back pain, and vomiting; hematologic adverse events included thrombocytopenia, neutropenia, anemia, leukopenia, and lymphopenia.

**Conclusions:**

This study indicates that venetoclax alone or in combination with other agents reveals favorable treatment responses and acceptable adverse events in treating RRMM.

**Supplementary Information:**

The online version contains supplementary material available at 10.1186/s12885-023-11553-3.

## Background

Multiple myeloma (MM) is an incurable hematologic malignancy characterized by relapses and remissions [1]. Generally, clinical symptoms of MM include hypercalcemia, elevated serum creatinine, renal insufficiency, anemia, and bone destruction [2, 3]. With a global incidence of approximately 4/100,000 per year, MM accounts for about 10% of all hematological malignancies [4–6]. Until 2000, the standard therapy for MM was melphalan or doxorubicin-based regimens with corticosteroids. Applying proteasome suppressants, histone deacetylase inhibitors, immunomodulatory agents, and monoclonal antibodies has posed various treatment choices for MM patients. Unfortunately, most MM patients eventually relapse and become resistant to different therapies with a decreased response rates to the regimens in subsequent recurrences [7]. It is widely deemed that genomic instability, clonal heterogeneity, and myeloma microenvironment interactions are pivotal causes of treatment resistance and relapse [1, 8]. Antiapoptotic BCL-2 family proteins are key in regulating the internal apoptotic pathway and cell survival [9–11]. It has been proven that BCL-2 is overexpressed in myeloma cell subsets and participates in their survival [12]. Analysis of BCL-2 homologous domain three recently confirmed the role of BCL-2 in maintaining MM cell survival [13].

Moreover, the selective inhibition of BCL-2 restores the apoptotic pathway of malignant cells [14–16]. Venetoclax, an oral, potent inhibitor of BCL-2, has been observed to show antitumor activity in many hematological malignancies [14–16]. It has been proven to induce apoptosis in human MM cell lines and original samples of MM patients, especially those cells carrying t(11; 14) chromosome translocation [17]. In addition, venetoclax as a monotherapy or in combination with other agents showed significant clinical activity against relapsed/refractory MM (RRMM), particularly in patients with t(11;14) [18–20]. Combining venetoclax and agents with complementary action mechanisms (such as IMiDs) or agents that increase BCL-2 dependency may enhance the anti-MM activity of venetoclax [21]. This meta-analysis aimed to assess the efficacy and safety of venetoclax and the combined therapy with other agents by synthesizing the results from published articles.

## Methods

This meta-analysis was done based on previously published studies that had declared ethical approvals, thereby ethical approval was not required for this study. This study was based on the Preferred Reporting Items for Systematic Reviews and Meta-analysis (PRISMA) [22].

### Search strategy and selection criteria

We searched the electronic databases, including PubMed, Web of Science, Embase, and Cochrane Library, up to November 7, 2022, with citations in English. The following key terms were used: BCL-2, venetoclax, and multiple myeloma. The detailed search strategy is shown in Supplementary Table 1. The references of included articles were also searched to assay additional studies. Inclusion criteria were: (1) clinical trials investigating the efficacy and adverse events of venetoclax or venetoclax with other agents in patients with RRMM. (2) Outcomes regarding treatment responses and adverse events could be extracted or calculated. (3) If studies recruited participants over the same period or from the same center, we only included the study with the largest sample size. We excluded case reports, reviews, comments, editorials, and conference abstracts with unavailable indicators. Two independent investigators (Xiaohui Gao and Xiaoyan Zhao) performed a literature search and study inclusion. When disagreement occurred, they discussed their arguments, and a third reviewer (Gang Zhang) was involved when no consensus was achieved.

### Data extraction and quality assessments

Two reviewers independently screened the title and abstract according to the inclusion criteria. Then a full-text reading of the literature was performed for the final identification for eligibility. The following information was extracted: name of the first author, year of publication, study design, number of patients, age, treatment regimens, the dosage of venetoclax, percentage of patients positive for t(11;14), and treatment outcomes. The outcomes comprised overall response rates (ORR), stringent complete response rates (sCR), complete response rates (CR), very good partial response rates (VGPR), partial response rates (PR), stable disease (SD), progressive disease (PD) as well as adverse events. We assessed the quality of the enrolled studies using Methodological Index for Non-Randomized Studies (MINORS).

### Statistical analysis

We used the R studio (Version 3.6.1; A language and environment for statistical computing. R Foundation for Statistical Computing, Vienna, Austria) for statistical analyses. A Cochran Q test and I² statistic were used to investigate heterogeneity [23]. The pooled ORR, sCR, CR, VGPR, PR, SD, PD, and adverse events rates with their respective 95% confidence intervals (CIs) were calculated using a random or fixed-effects model. A random-effects model was used if the I² value was > 50%. Otherwise, a fixed-effects model was used [24]. Subgroup analysis and meta-regression were performed based on population baselines, including study design, age, sample size, regimen, the dose of venetoclax, and t(11;14) status. We conducted a sensitivity analysis to check the stability of pooled outcomes. Furthermore, Egger’s tests were performed to assess the potential publication bias. A probability of P < 0.05 was regarded as statistically significant.

## Results

### Study selection and characteristics

**Fig. 1:**
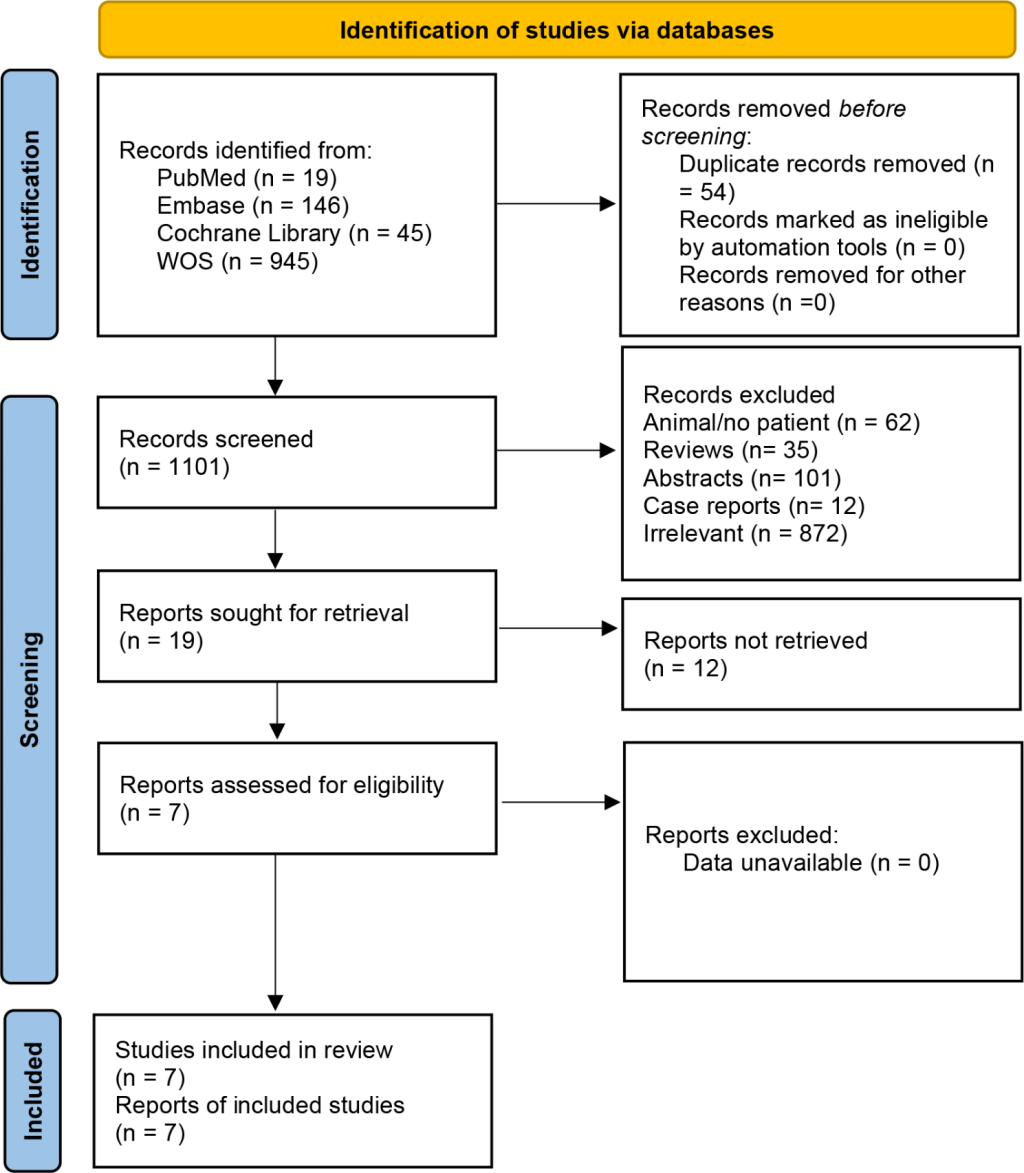
Search results and flow chart of the meta-analysis

We identified 1155 articles from the databases searched. Afterward, 54 duplicates were removed, and 992 studies were excluded through an initial screening. After a full-text assessment of the remaining 19 articles, seven studies were identified for inclusion [18–21, 25–27] (Fig. 1). The selected seven studies containing 482 patients with diagnosed RRMM provided the outcomes needed in this study. Six trials reported the efficacy and safety of venetoclax in combination with other agents, and one trial investigated the efficacy and safety of venetoclax monotherapy. The quality of included studies was rated as moderate to high according to the MINORS tool. Table 1 shows detailed information on included studies.

**Table 1:** Study characteristics

Author	Year	Study design	No. of patients	Age, median (range), y	Regimens	Dosage of venetoclax	Positive for t(11;14) (%)	Prior lines of therapy, median (range)	Refractory, n (%)	OS, PFS	MINORS scores
Kumar	2017	Open-label, phase 1 study	66	63 (31–79)	Monotherapy	21-day cycles with daily venetoclax given at final doses of 300, 600, 900, or 1200 mg in dose-escalation cohorts (3 + 3 design) and 1200 mg in the safety expansion	46	5 (1–15)	Bortezomib:46 (70); Lenalidomide: 51 (77); Bortezomib/lenalidomide: 40 (61); Carfilzomib: 20(30); Pomalidomide: 35 (53)	NR	13
Moreau	2018	Open-label, multicenter, phase 1b	66	64 (38–79)	Venetoclax in combination with bortezomib and dexamethasone	Once daily (100, 200, 300, 400, 500, 600, 800, 1000, and 1200 mg)	14	3 (1–13)	Bortezomib: 26 (39); lenalidomide: 35 (53)	NR	14
Kumar	2020	Randomised, doubleblind, placebocontrolled, multicentre, phase 3 trial	194	66	Venetoclax plus bortezomib and dexamethasone	800 mg orally daily	10	> 1 line:53%	Immunomodulatory drugs: 64 (33); lenalidomide: 38(20)	Median PFS was 22.4 months (95% CI 15.3–not estimable) with venetoclax versus 11.5 months (9.6–15.0) with placebo. 41 (21%) overall survival events occurred in the venetoclax group compared with 11 (11%) in the placebo group.	14
Bahlis	2021	Multicenter, phase 1	48	64 (41–80)	Venetoclax plus daratumumab and dexamethasone	Once daily at 400 mg	63	Part 1:2.5 (1–8); Part 2:1 (1–3)	PI: 11 (23); IMiD: 25 (52); PI + IMiD: 10 (21)	The 18-month PFS rate was 90.5% (95% CI, 67.0 to 97.5) with venetoclax + daratumumab + dexamethasone and 66.7% (95% CI, 42.5 to 82.5) with venetoclax + daratumumab + bortezomib + dexamethasone	14
Costa	2021	Open-label, multicenter, phase 2	49	66 (37–79)	Venetoclax plus carfilzomib and dexamethasone	Daily (400 or 800 mg)	27	1 (1–3)	PI: 28 (57); IMiD: 35 (71); PI + IMiD: 22 (45)	Median PFS was 22.8 months	13
Gasparetto	2021	Open-label, multicenter, phase 2	8	68 (60–77)	Venetoclax in combination with pomalidomide and dexamethasone	Orally at 400 mg daily	38	> 2 lines:50%	PI: 2 (25); lenalidomide: 6 (75); daratumumab: 4 (50); PI + lenalidomide: 2(25); PI + lenalidomide + daratumumab: 2(25)	OS at 6 months was 87.5%	14
Kaufman	2021	Open-label phase 1/2 study	51	46–80	Venetoclax and dexamethasone	800 mg on days 1, 8, and 15	100	Part 1:3 (1–8); Part 2:5 (2–12)	PI: 40 (78); IMiD: 45 (88); PI + IMiD: 31 (61); daratumumab: 31 (61)	The median time to progression was 12.4/10.8 months.	15

OS, overall survival. PFS, progression free survival. MINORS: methodological index for non-randomized studies. PI, proteasome inhibitor

IMiD: immunomodulatory imide drug

### Efficacy

**Fig. 2:**
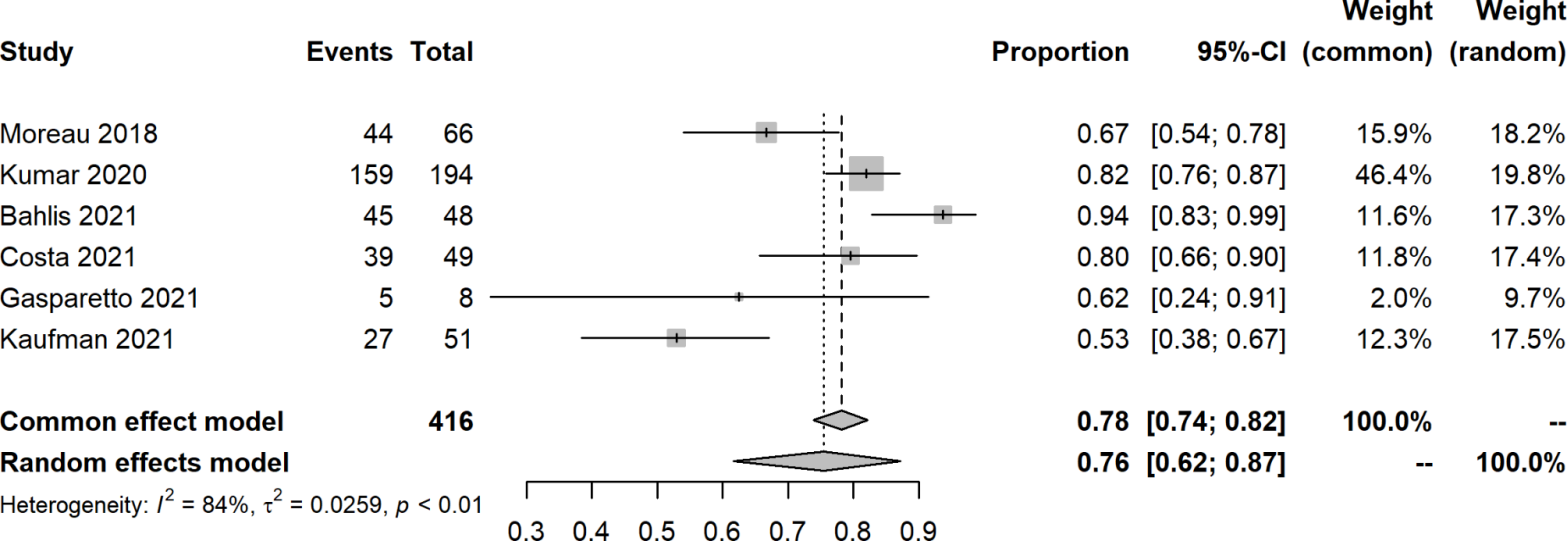
Forest plot of overall response rates

**Fig. 3:**
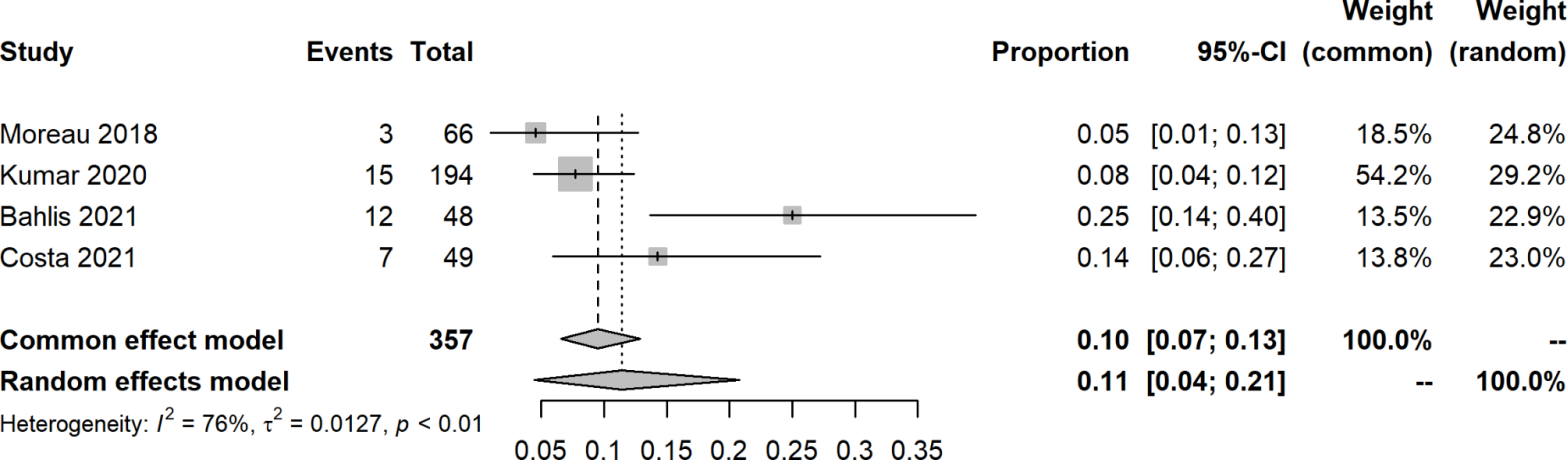
Forest plot of stringent complete response rates

**Fig. 4:**
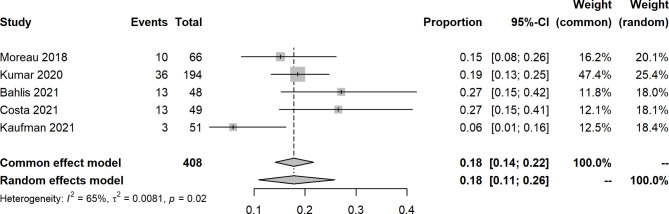
Forest plot of complete response rates

**Fig. 5:**
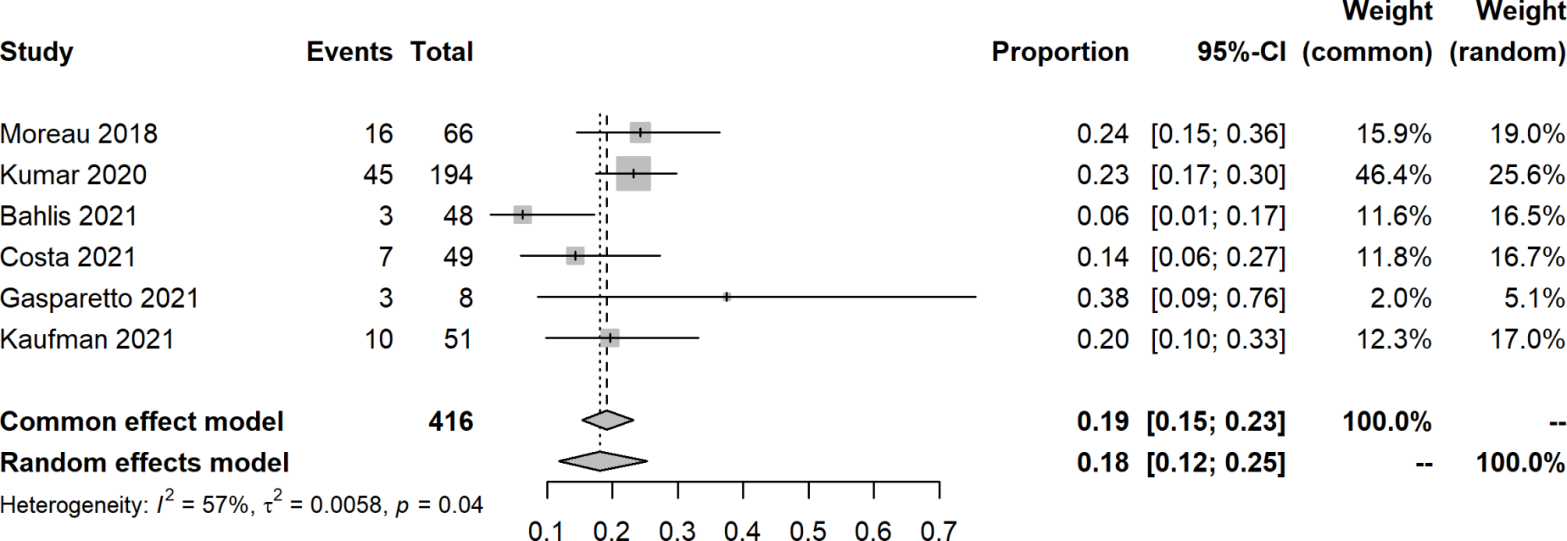
Forest plot of very good partial response rates

**Fig. 6:**
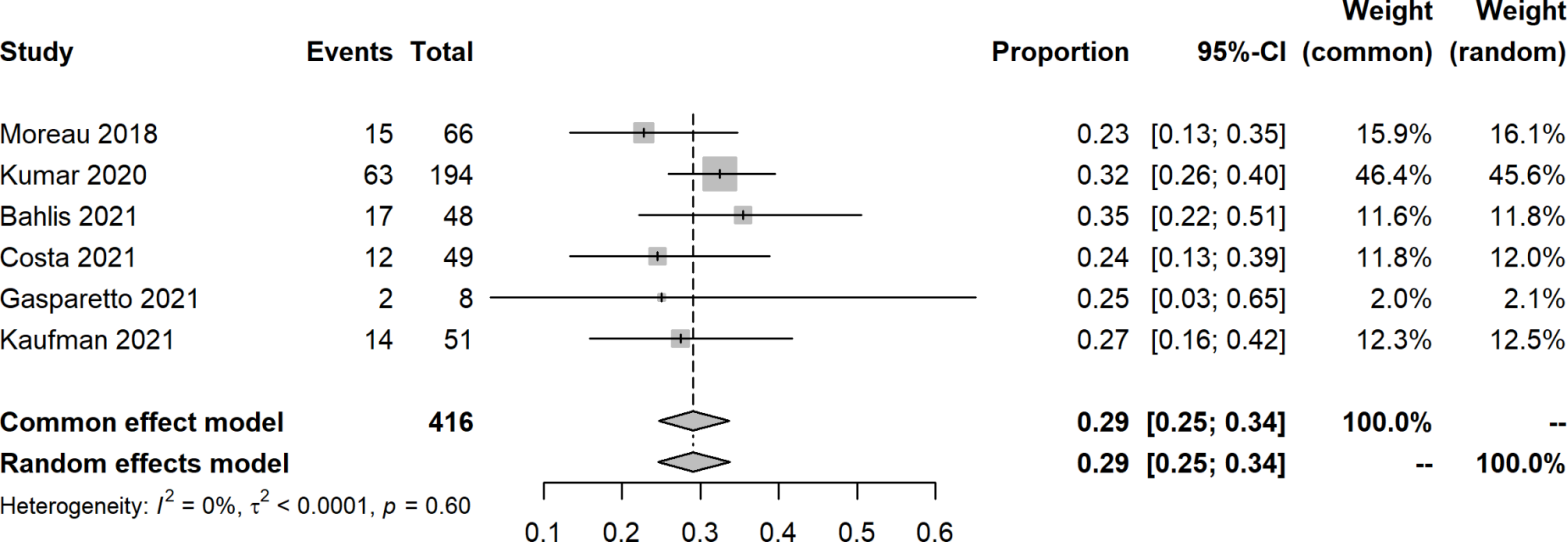
Forest plot of partial response rates

An open-label, dose-escalation, phase 1 study of venetoclax monotherapy in 66 patients with RRMM revealed that the ORR was 0.21 (14/66), and 15% achieved VGPR or better. Most responses (12/14) were reported in patients with t(11;14). For included studies about venetoclax with other agents, the pooled ORR, sCR, CR, VGPR, PR, SD, and PD were 0.76 (95% CIs: 0.62, 0.87; I^2^ = 84%, p < 0.0001), 0.11 (95% CIs: 0.04, 0.21; I^2^ = 77%, p = 0.0052), 0.18 (95% CIs: 0.11, 0.26; I^2^ = 65%, p = 0.0209), 0.16 (95% CIs: 0.12, 0.25; I^2^ = 57%, p = 0.0388), 0.29 (95% CIs: 0.25, 0.34; I^2^ = 0%, p = 0.5967), 0.07 (95% CIs: 0.05, 0.10; I^2^ = 0%, p = 0.4423), and 0.11 (95% CIs: 0.04, 0.23; I^2^ = 85%, p = 0.0015), respectively (Figs. 2, 3, 4, 5 and 6, **Figures ****S1****-****S2**). In a 2-phase study by Kaufman et al., t(11;14), only patients were recruited; the ORR was 60% and 48% in phase 1 and phase 2, respectively.

Median progression-free survival ranged from 22.4 to 22.8 months. Gasparetto’s study reported overall survival at six months of 87.5%. The 18-month PFS rate was 90.5% (95% CI, 67.0 to 97.5) with venetoclax + daratumumab + dexamethasone and 66.7% (95% CI, 42.5 to 82.5) with venetoclax + daratumumab + bortezomib + dexamethasone in the trial of Bahlis et, al.

### Adverse events

In the included study of venetoclax monotherapy, the most common adverse events had mild gastrointestinal symptoms (nausea [47%], diarrhea [36%], and vomiting [21%]). Cytopenias were the most common grade 3/4 events, with thrombocytopenia (32%), neutropenia (27%), anemia (23%), and leukopenia (23%) reported. For included studies investigating venetoclax with other agents, the overall rate of adverse events ≥ Grade 3 was 0.84 (95% CIs: 0.77, 0.91; I^2^ = 61%, p = 0.0235) (Figure S3). The most common non-hematologic adverse event included nausea (0.38), diarrhea (0.53), fatigue (0.33), back pain (0.18), and vomiting (0.19) (Table 2, Figures S4-S8). Hematologic adverse events included thrombocytopenia (0.25), neutropenia (0.21), anemia (0.22), leukopenia (0.20), and lymphopenia (0.26) (Table 2, Figures S9-S13).

**Table 2:** Summary of adverse events

Adverse event	Pooled rates of any grade	95% CIs	I^2^ (%)	p
Non-hematologic adverse events				
Nausea	0.38	0.33–0.42	34	0.1798
Diarrhea	0.53	0.42–0.63	69	0.0129
Fatigue	0.33	0.21–0.46	78	0.0005
Back pain	0.18	0.13–0.24	0	0.6993
Vomiting	0.19	0.14–0.23	29	0.2372
**Hematologic adverse events**				
Thrombocytopenia	0.25	0.17–0.34	64	0.0171
Neutropenia	0.21	0.17–0.26	58	0.0347
Anemia	0.22	0.18–0.26	49	0.0831
Leukopenia	0.20	0.13–0.28	8	0.3384
Lymphopenia	0.26	0.20–0.34	3	0.3774

CI: confidence interval

### Publication bias

Egger’s tests for publication bias revealed p-values of 0.5348, 0.3949, 0.9958, 0.7642, 0.4547, 0.1241, 0.3522, and 0.9626 for the analyses of ORR, sCR, CR, VGPR, PR, SD, PD, and adverse events rates ≥ Grade 3, respectively.

### Sensitivity analysis

The pooled outcomes showed robustness in sensitivity analysis with the leave-one-out method (Figures S14-S21).

## Discussion

In the past two decades, there were dramatic advances in the treatment of MM, beginning with the reported use of high-dose melphalan and autologous stem cell transplant in 1996 [28], followed by the introduction of the immunomodulatory drugs [29], the proteasome inhibitors (PI) [30] and BCL-2 inhibitor [31]. Those drugs are active in RRMM and expanded the treatment options for patients. This meta-analysis included seven published articles comprising 482 patients with diagnosed RRMM and treated with venetoclax-based regimens. For venetoclax monotherapy, Kumar’s phase 1 trial showed an ORR of 0.21. For included studies investigating venetoclax with other agents, the pooled ORR, sCR, CR, VGPR, PR, SD, and PD were 0.76, 0.11, 0.18, 0.16, 0.29, 0.07, and 0.11. Median PFS ranged from 22.4 to 22.8 months. Gasparetto’s study reported overall survival at six months of 87.5%. In the Bahlis et al. trial, venetoclax + daratumumab + dexamethasone had a higher 18-month PFS rate compared to venetoclax + daratumumab + bortezomib + dexamethasone [25]; and an increased rate of fatal infections was observed in patients treated with venetoclax + bortezomib + dexamethasone in the phase 3 BELLINI trial [20]. The combination of different drugs may affect drug metabolism, leading to differences in efficacy and safety. It may be one of the underlying causes of the diversity in PFS, which needs further investigation.

Preclinical studies have shown that both the dexamethasone and the proteasome inhibitors (bortezomib and carfilzomib) can increase BCL­2 dependency in MM cells by shifting MCL­1 to BCL­2 and by decreasing MCL­1 activity through the upregulation of Noxa (PMAIP1) [32–36]. Furthermore, daratumumab, a CD38 monoclonal antibody, can induce cellular death in MM cells through complement-dependent cytotoxicity, antibody-dependent cytotoxicity, antibody-dependent cellular phagocytosis with an expansion of clonal effector T cells, and decrease of regulatory T cells and, therefore, could eliminate emergent resistant subclones [37]. Venetoclax, in turn, was demonstrated to enhance adaptive immunity by increasing the CD4 + and CD8 + effector memory cells in the blood and improving the efficacy of immune checkpoint blockade [38]. In addition, a combination of venetoclax and pomalidomide was proven to increase immune stimulation [38, 39]. Regarding adverse events, the most common non-hematologic adverse events were nausea, diarrhea, fatigue, back pain, and vomiting. Hematologic adverse events included thrombocytopenia, neutropenia, anemia, leukopenia, and lymphopenia. The pooled rate of adverse events ≥ Grade 3 was 0.84.

In this meta-analysis, there were significant heterogeneities in all indicators. The heterogeneity may be attributed to differences in baseline characteristics of the study participants, study design, drug compliance, median lines of prior therapy in each study, and other relevant factors. Regardless, sensitivity analyses demonstrated the robustness of the results of this meta-analysis, and Egger’s publication test also showed no significant publication bias in the included studies.

We acknowledge additional limitations of this study: firstly, the heterogeneity existed on venetoclax doses and combinations in included studies, which subgroup analysis could not address due to a limited number of studies in each subgroup. Secondly, the included studies were single-armed clinical trials, or data from one arm of one randomized controlled clinical trial was analyzed. These trials were limited by the lack of a randomized design, not to mention the inherent limitations of cross-trial comparisons. Finally, the subgroup of patients with multiple myeloma with or without t(11;14) was not performed because the data from included studies relevant to these subgroups could not be extracted. Individual patient data are warranted to address this issue. Despite limitations in this meta-analysis, the results provide a pooled analysis of the efficacy and safety of venetoclax with a large sample size and a comprehensive description and quality assessment of the relevant clinical trial profiles. This study addresses gaps in the existing evidence and supports future clinical trials with a different focus.

## Conclusions

Based on the outcomes of this meta-analysis, we may conclude that venetoclax combined with other agents has promising clinical response rates in treating RRMM patients who received at least one line of prior therapy, with acceptable adverse effects. It is expected that well-designed randomized controlled clinical trials and real-world studies be conducted to address issues in evaluating the efficacy and safety of venetoclax monotherapy or in combination with novel agents in treating RRMM.

### Electronic supplementary material

Below is the link to the electronic supplementary material.

Supplementary Material 1

Supplementary Material 2

## Data Availability

All data generated or analyzed during this study are included in this article and its supplementary information file.

## List of abbreviations

RRMM: Relapsed/refractory multiple myeloma
ORR: Overall response rates
sCR: Stringent complete response rates
CR: Complete response
VGPR: Very good partial response rates
PR: Partial response
SD: Stable disease
PD: Progressive disease
MM: Multiple myeloma
MGUS: Monoclonal gammopathy of uncertain significance
PRISMA: Preferred Reporting Items for Systematic Reviews and Meta-analysis
MINORS: Methodological Index for Non-Randomized Studies
CIs: Confidence intervals
PI: Proteasome inhibitors

## References

[1] Nair R, Patel K (2018). Emerging role of CAR T cell therapy in Multiple Myeloma. Adv Cell Gene Therapy.

[2] Michels TC, Petersen KE (2017). Multiple Myeloma: diagnosis and treatment. Am Fam Physician.

[3] Kehrer M, Koob S, Strauss A, Wirtz DC, Schmolders J (2017). Multiple Myeloma - current Status in Diagnostic Testing and Therapy. Z Orthop Unfall.

[4] Rajkumar SV, Kumar S (2016). Multiple Myeloma: diagnosis and treatment. Mayo Clin Proc.

[5] Palumbo A, Bringhen S, Ludwig H, Dimopoulos MA, Blade J, Mateos MV (2011). Personalized therapy in Multiple Myeloma according to patient age and vulnerability: a report of the European Myeloma Network (EMN). Blood.

[6] Kriegsmann K, Kriegsmann M, Cremer M, Schmitt M, Dreger P, Goldschmidt H (2019). Cell-based immunotherapy approaches for Multiple Myeloma. Br J Cancer.

[7] van de Donk N, Pawlyn C, Yong KL (2021). Multiple Myeloma. Lancet.

[8] Wu C, Zhang L, Brockman QR, Zhan F, Chen L (2019). Chimeric antigen receptor T cell therapies for Multiple Myeloma. J Hematol Oncol.

[9] Green DR, Walczak H (2013). Apoptosis therapy: driving cancers down the road to ruin. Nat Med.

[10] Scarfò L, Ghia P (2013). Reprogramming cell death: BCL2 family inhibition in hematological malignancies. Immunol Lett.

[11] Delbridge AR, Grabow S, Strasser A, Vaux DL (2016). Thirty years of BCL-2: translating cell death discoveries into novel cancer therapies. Nat Rev Cancer.

[12] Bodet L, Gomez-Bougie P, Touzeau C, Dousset C, Descamps G, Maïga S (2011). ABT-737 is highly effective against molecular subgroups of Multiple Myeloma. Blood.

[13] Touzeau C, Ryan J, Guerriero J, Moreau P, Chonghaile TN, Le Gouill S (2016). BH3 profiling identifies heterogeneous dependency on Bcl-2 family members in Multiple Myeloma and predicts sensitivity to BH3 mimetics. Leukemia.

[14] Konopleva M, Pollyea DA, Potluri J, Chyla B, Hogdal L, Busman T (2016). Efficacy and Biological correlates of response in a phase II study of Venetoclax Monotherapy in patients with Acute Myelogenous Leukemia. Cancer Discov.

[15] Roberts AW, Davids MS, Pagel JM, Kahl BS, Puvvada SD, Gerecitano JF (2016). Targeting BCL2 with Venetoclax in Relapsed Chronic lymphocytic Leukemia. N Engl J Med.

[16] Stilgenbauer S, Eichhorst B, Schetelig J, Coutre S, Seymour JF, Munir T (2016). Venetoclax in relapsed or refractory chronic lymphocytic Leukaemia with 17p deletion: a multicentre, open-label, phase 2 study. Lancet Oncol.

[17] Touzeau C, Dousset C, Le Gouill S, Sampath D, Leverson JD, Souers AJ (2014). The Bcl-2 specific BH3 mimetic ABT-199: a promising targeted therapy for t(11;14) Multiple Myeloma. Leukemia.

[18] Kaufman JL, Gasparetto C, Schjesvold FH, Moreau P, Touzeau C, Facon T (2021). Targeting BCL-2 with venetoclax and dexamethasone in patients with relapsed/refractory t(11;14) Multiple Myeloma. Am J Hematol.

[19] Kumar S, Kaufman JL, Gasparetto C, Mikhael J, Vij R, Pegourie B (2017). Efficacy of venetoclax as targeted therapy for relapsed/refractory t(11;14) Multiple Myeloma. Blood.

[20] Kumar SK, Harrison SJ, Cavo M, de la Rubia J, Popat R, Gasparetto C (2020). Venetoclax or placebo in combination with bortezomib and dexamethasone in patients with relapsed or refractory Multiple Myeloma (BELLINI): a randomised, double-blind, multicentre, phase 3 trial. Lancet Oncol.

[21] Gasparetto C, Bowles KM, Abdallah AO, Morris L, Mander G, Coppola S (2021). A phase II study of Venetoclax in Combination with Pomalidomide and Dexamethasone in Relapsed/Refractory Multiple Myeloma. Clin Lymphoma Myeloma Leuk.

[22] Moher D, Liberati A, Tetzlaff J, Altman DG (2010). Preferred reporting items for systematic reviews and meta-analyses: the PRISMA statement. Int J Surg.

[23] Dziri C (2022). How to assess heterogeneity for a meta-analysis?. Tunis Med.

[24] Tao Y, Zhou H, Niu T (2021). Safety and Efficacy Analysis of Selinexor-based treatment in Multiple Myeloma, a Meta-analysis based on prospective clinical trials. Front Pharmacol.

[25] Bahlis NJ, Baz R, Harrison SJ, Quach H, Ho SJ, Vangsted AJ (2021). Phase I study of Venetoclax Plus Daratumumab and Dexamethasone, with or without Bortezomib, in patients with relapsed or refractory Multiple Myeloma with and without t(11;14). J Clin Oncol.

[26] Costa LJ, Davies FE, Monohan GP, Kovacsovics T, Burwick N, Jakubowiak A (2021). Phase 2 study of venetoclax plus carfilzomib and dexamethasone in patients with relapsed/refractory Multiple Myeloma. Blood Adv.

[27] Moreau P, Chanan-Khan A, Roberts AW, Agarwal AB, Facon T, Kumar S (2017). Promising efficacy and acceptable safety of venetoclax plus bortezomib and dexamethasone in relapsed/refractory MM. Blood.

[28] Attal M, Harousseau JL, Stoppa AM, Sotto JJ, Fuzibet JG, Rossi JF (1996). A prospective, randomized trial of autologous bone marrow transplantation and chemotherapy in Multiple Myeloma. Intergroupe Francais Du Myelome. N Engl J Med.

[29] Singhal S, Mehta J, Desikan R, Ayers D, Roberson P, Eddlemon P (1999). Antitumor activity of thalidomide in refractory Multiple Myeloma. N Engl J Med.

[30] Richardson PG, Barlogie B, Berenson J, Singhal S, Jagannath S, Irwin D (2003). A phase 2 study of bortezomib in relapsed, refractory Myeloma. N Engl J Med.

[31] Sidiqi MH, Al Saleh AS, Kumar SK, Leung N, Jevremovic D, Muchtar E (2021). Venetoclax for the treatment of Multiple Myeloma: outcomes outside of clinical trials. Am J Hematol.

[32] Kuhn DJ, Chen Q, Voorhees PM, Strader JS, Shenk KD, Sun CM (2007). Potent activity of carfilzomib, a novel, irreversible inhibitor of the ubiquitin-proteasome pathway, against preclinical models of Multiple Myeloma. Blood.

[33] Qin JZ, Ziffra J, Stennett L, Bodner B, Bonish BK, Chaturvedi V (2005). Proteasome inhibitors trigger NOXA-mediated apoptosis in Melanoma and Myeloma cells. Cancer Res.

[34] Kervoëlen C, Ménoret E, Gomez-Bougie P, Bataille R, Godon C, Marionneau-Lambot S (2015). Dexamethasone-induced cell death is restricted to specific molecular subgroups of Multiple Myeloma. Oncotarget.

[35] Matulis SM, Gupta VA, Nooka AK, Hollen HV, Kaufman JL, Lonial S (2016). Dexamethasone treatment promotes Bcl-2 dependence in Multiple Myeloma resulting in sensitivity to venetoclax. Leukemia.

[36] Punnoose EA, Leverson JD, Peale F, Boghaert ER, Belmont LD, Tan N (2016). Expression Profile of BCL-2, BCL-XL, and MCL-1 predicts pharmacological response to the BCL-2 selective antagonist Venetoclax in Multiple Myeloma models. Mol Cancer Ther.

[37] van de Donk N, Usmani SZ (2018). CD38 antibodies in Multiple Myeloma: mechanisms of Action and modes of Resistance. Front Immunol.

[38] Kohlhapp FJ, Haribhai D, Mathew R, Duggan R, Ellis PA, Wang R (2021). Venetoclax increases Intratumoral Effector T cells and Antitumor Efficacy in Combination with Immune Checkpoint Blockade. Cancer Discov.

[39] Dredge K, Marriott JB, Todryk SM, Muller GW, Chen R, Stirling DI (2002). Protective antitumor immunity induced by a costimulatory thalidomide analog in conjunction with whole Tumor cell vaccination is mediated by increased Th1-type immunity. J Immunol.

