# Supplementary material for: Efficacy and safety of venetoclax in patients with relapsed/refractory multiple myeloma: a meta-analysis
Source: BMC Cancer. 2023 Nov 3;23:1058. doi: 10.1186/s12885-023-11553-3 (PMC10623759; doi:10.1186/s12885-023-11553-3)
Supplement: Supplementary file 2 — Supplementary Material 2 [file 12885_2023_11553_MOESM2_ESM.docx]

**
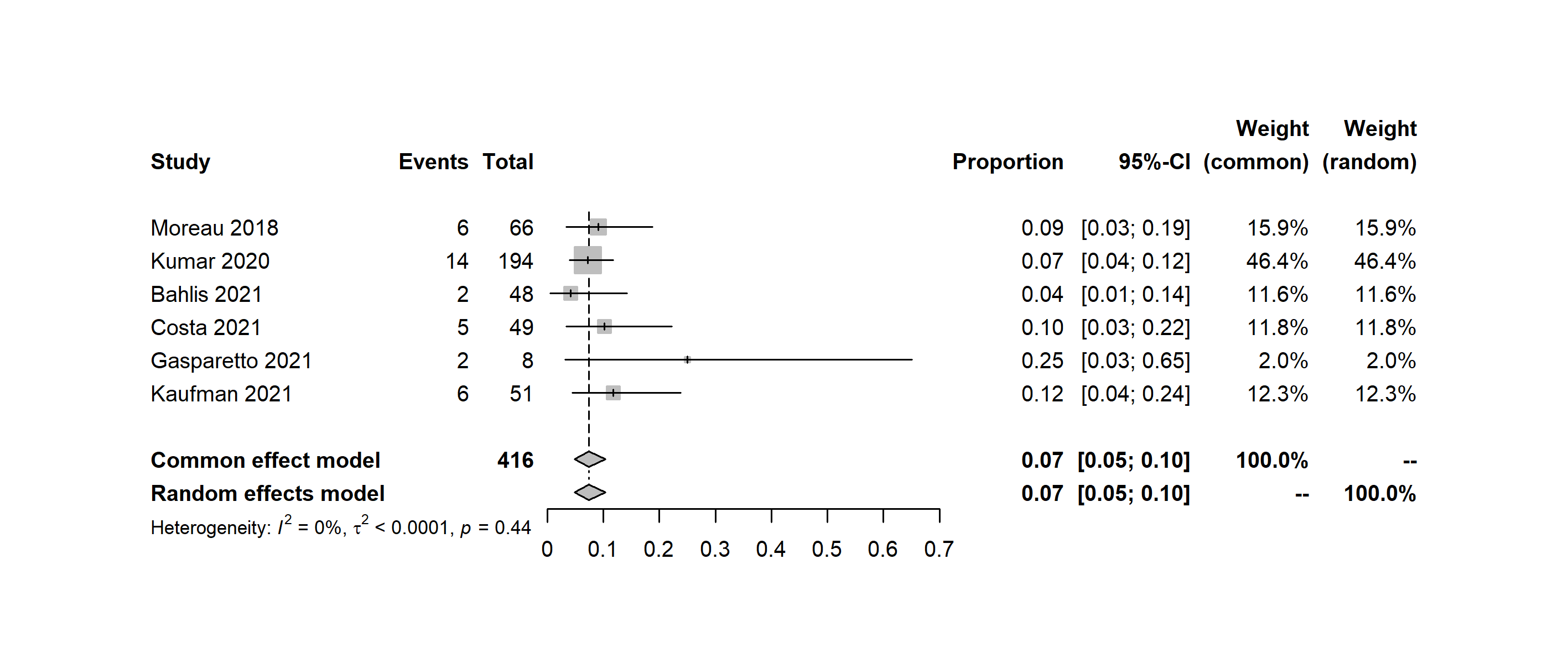
**

**Figure S1**. Forest plot of stable disease


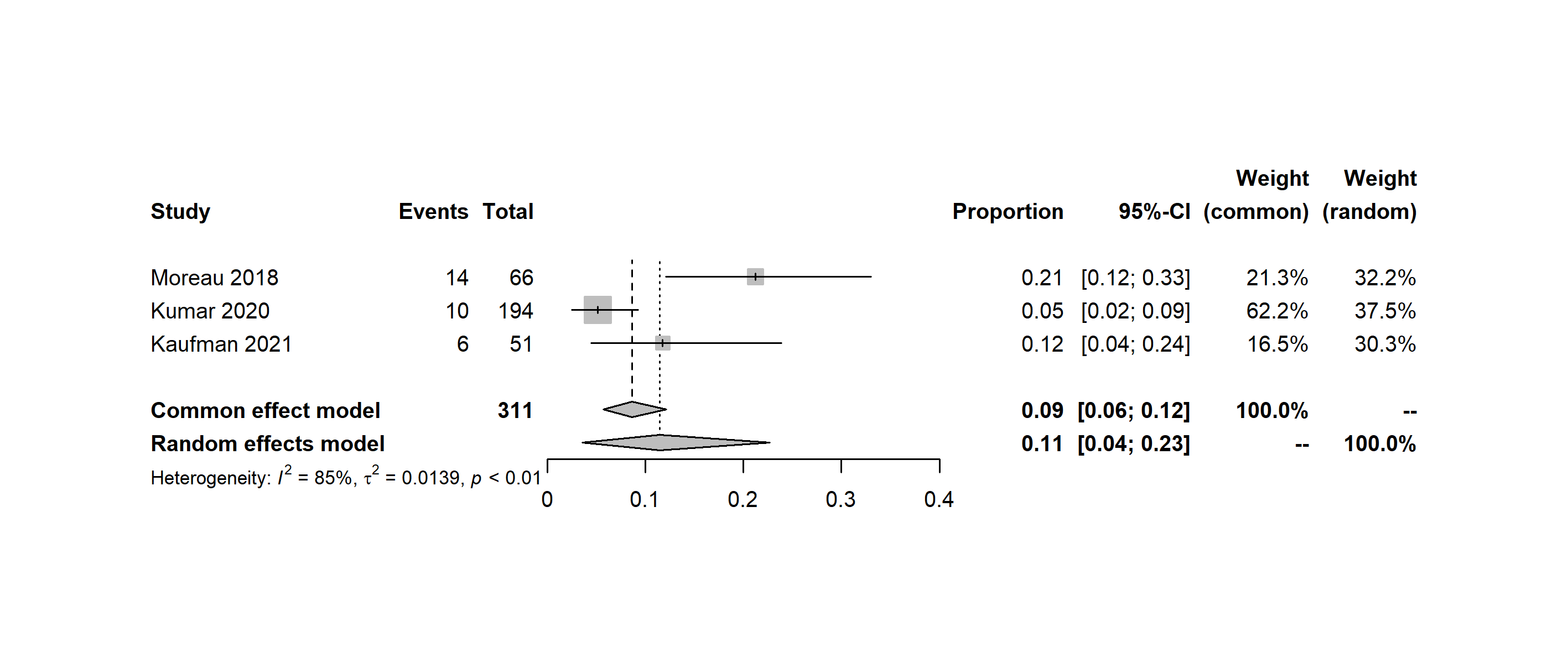


**Figure S2**. Forest plot of progressive disease


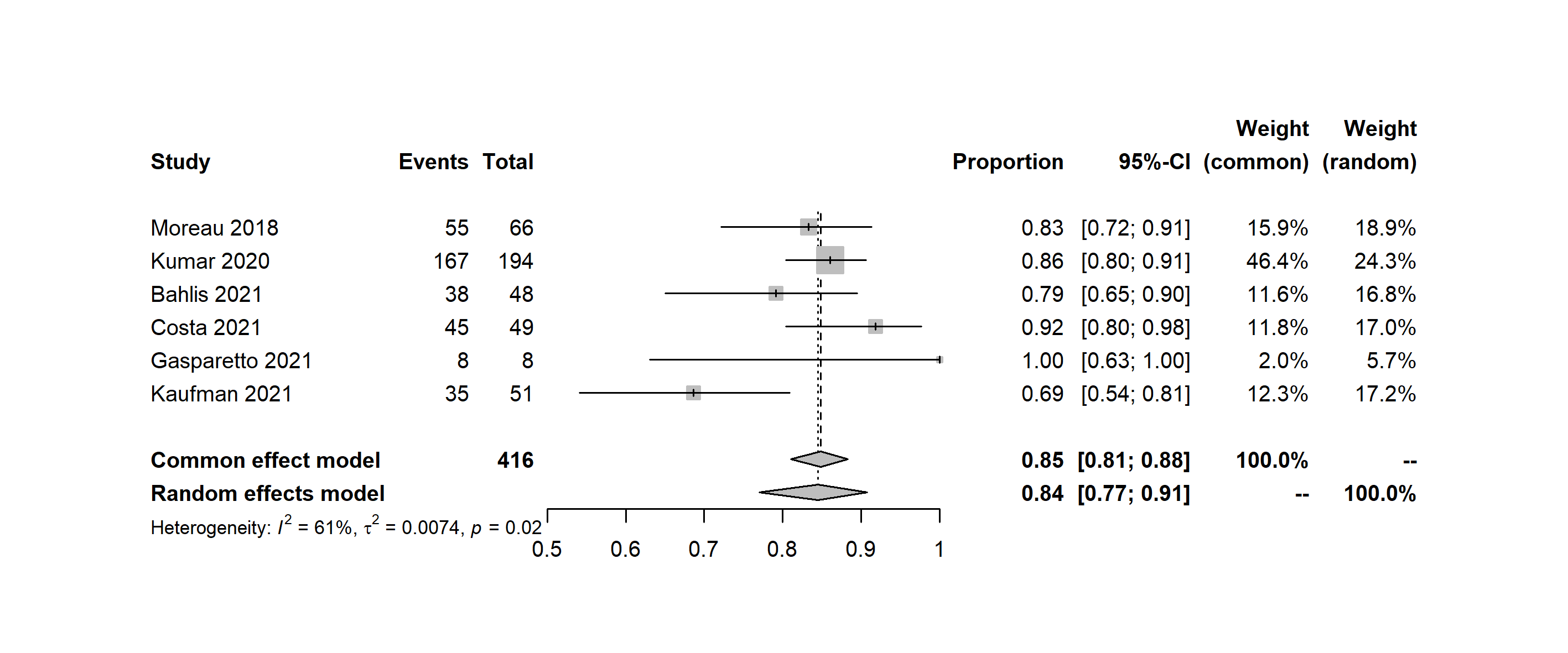


**Figure S3**. Forest plot of adverse events rates ≥ Grade 3


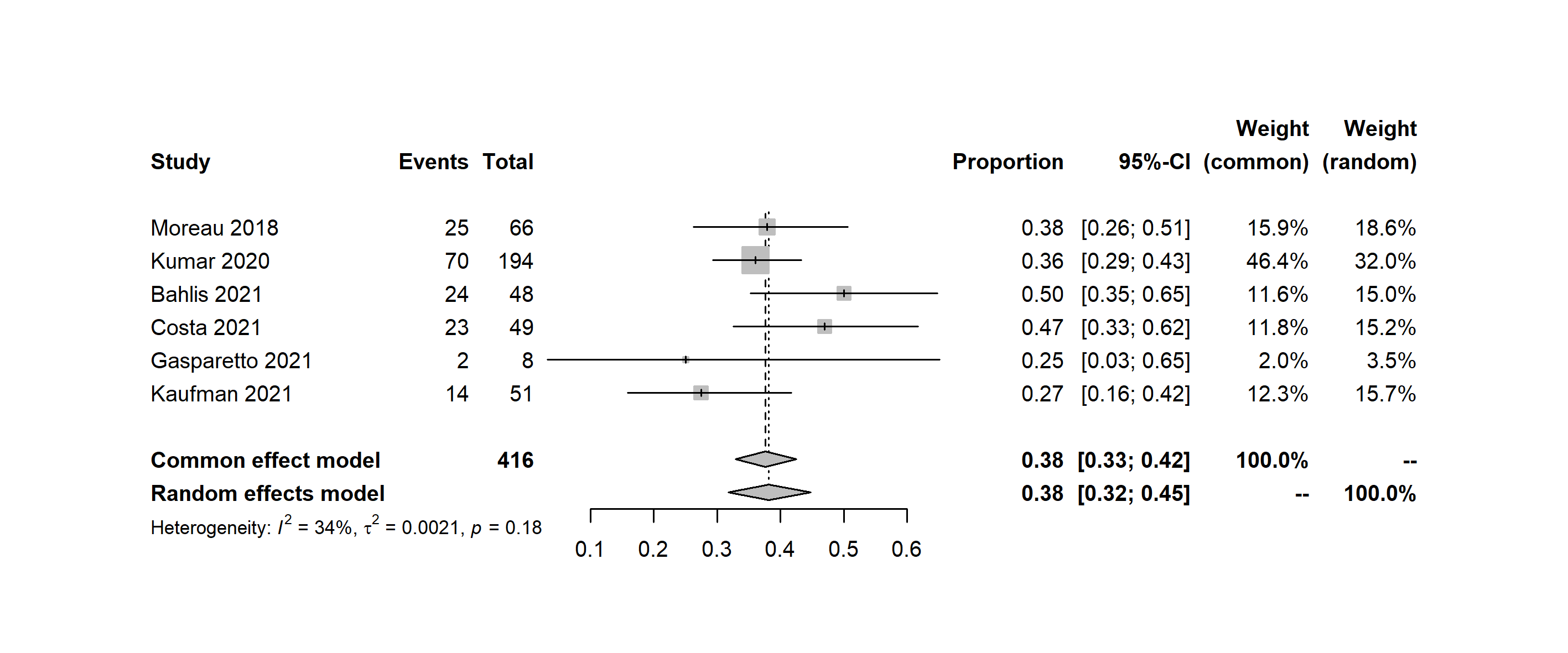


**Figure S4**. Forest plot of nausea


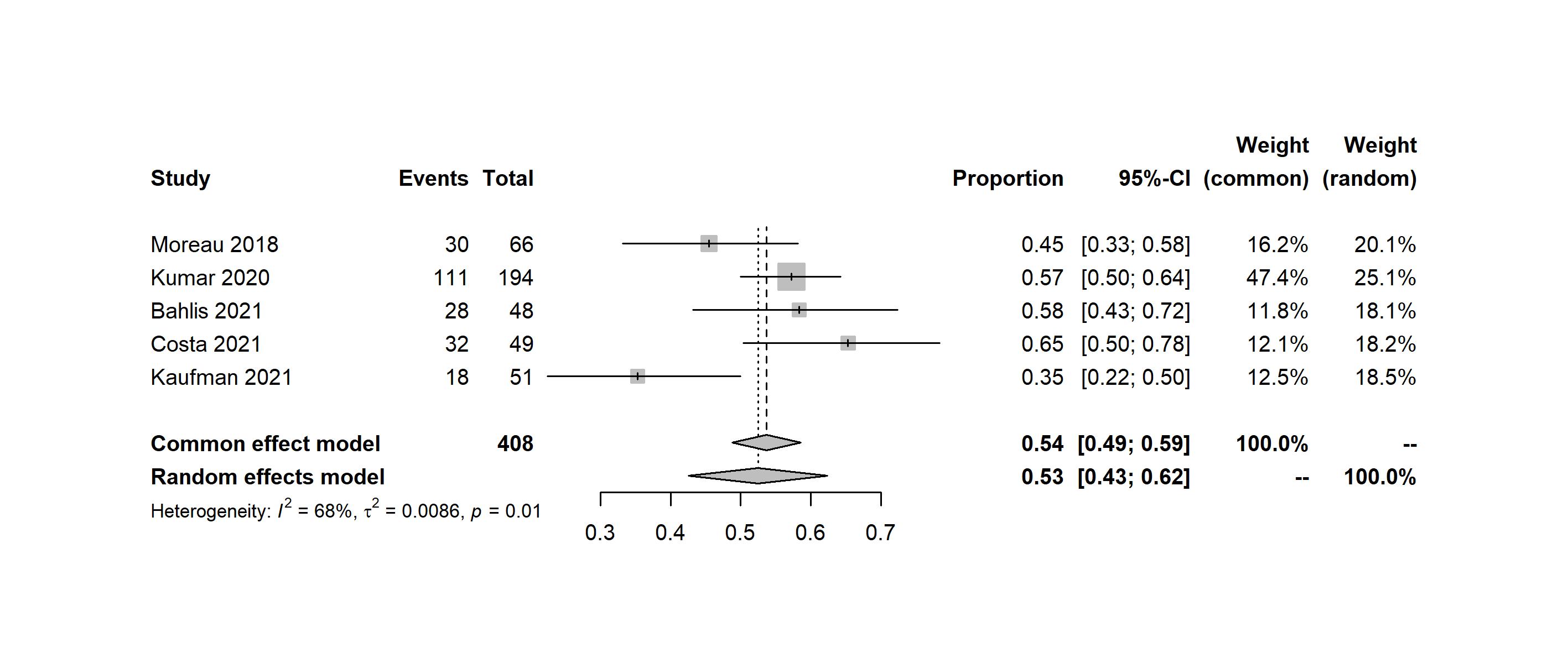
**Figure S5**. Forest plot of diarrhea


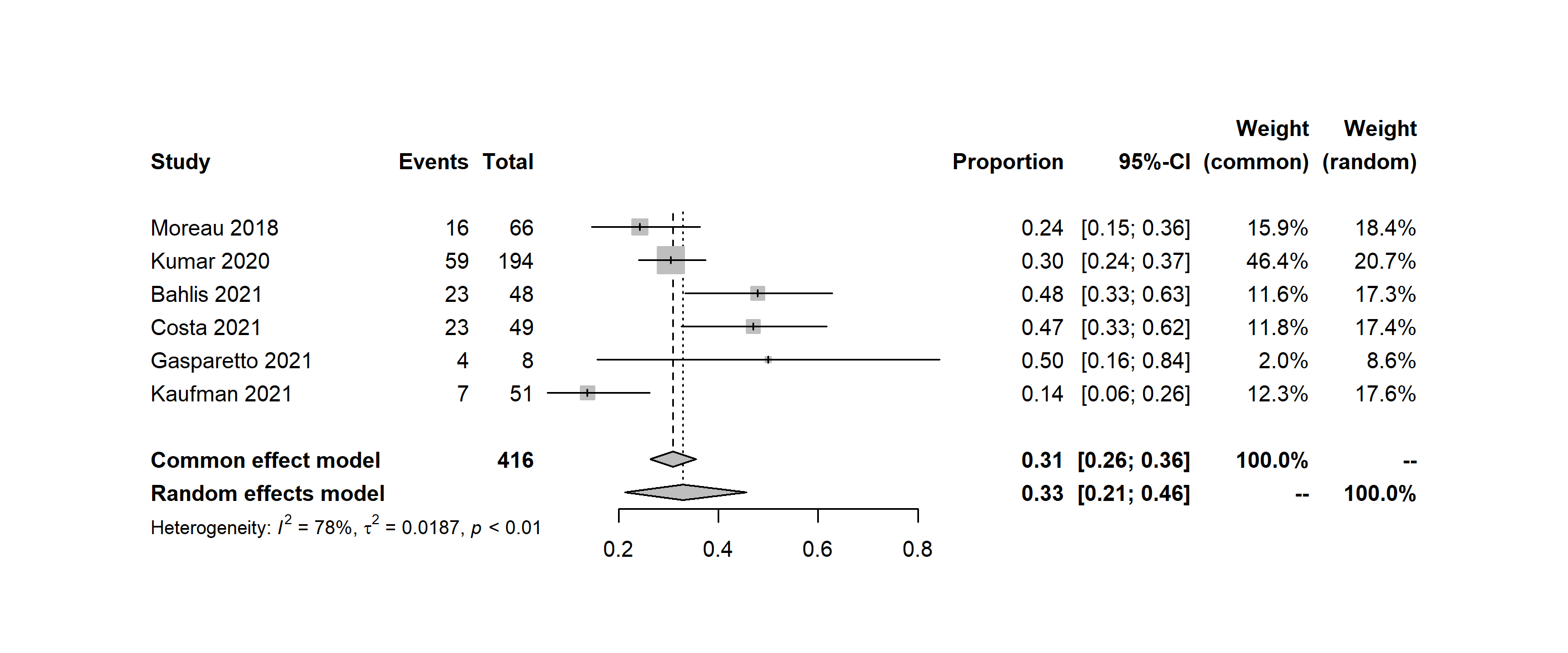
**Figure S6**. Forest plot of fatigue


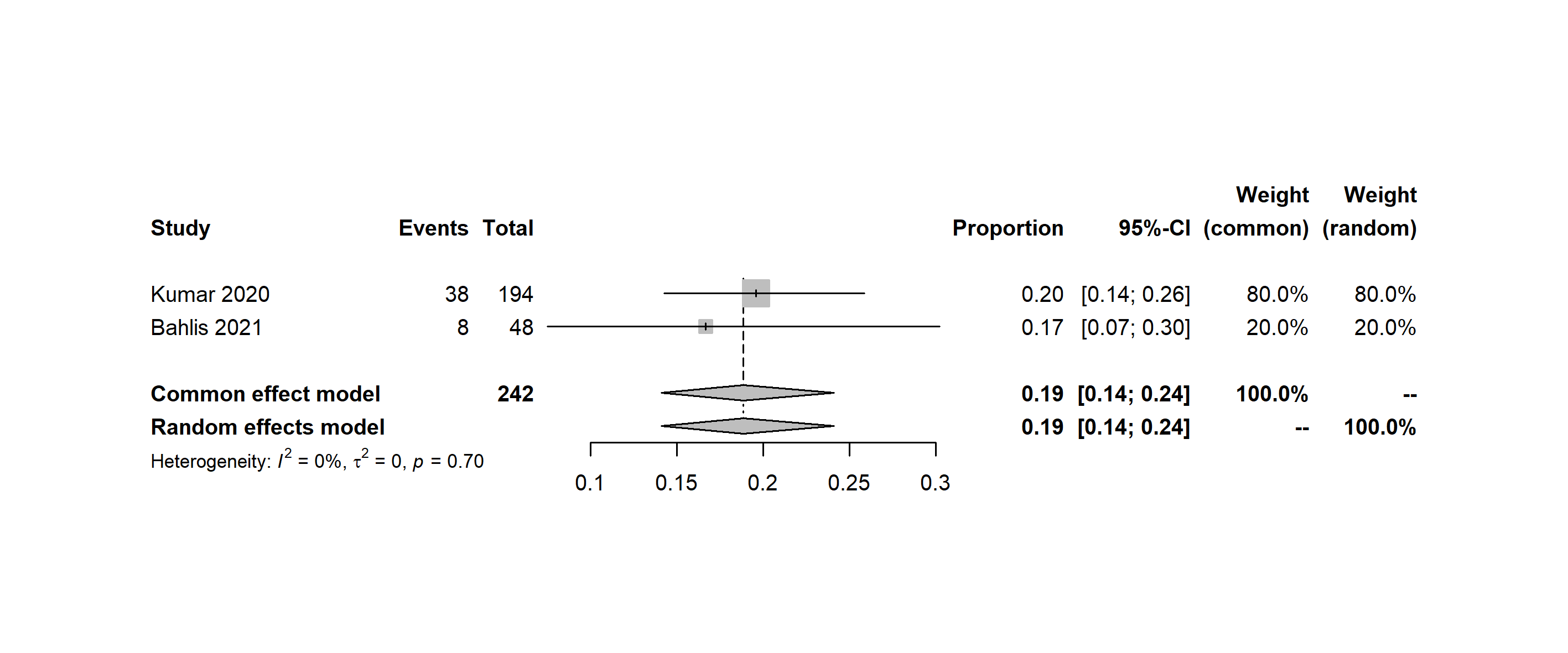
**Figure S7**. Forest plot of back pain


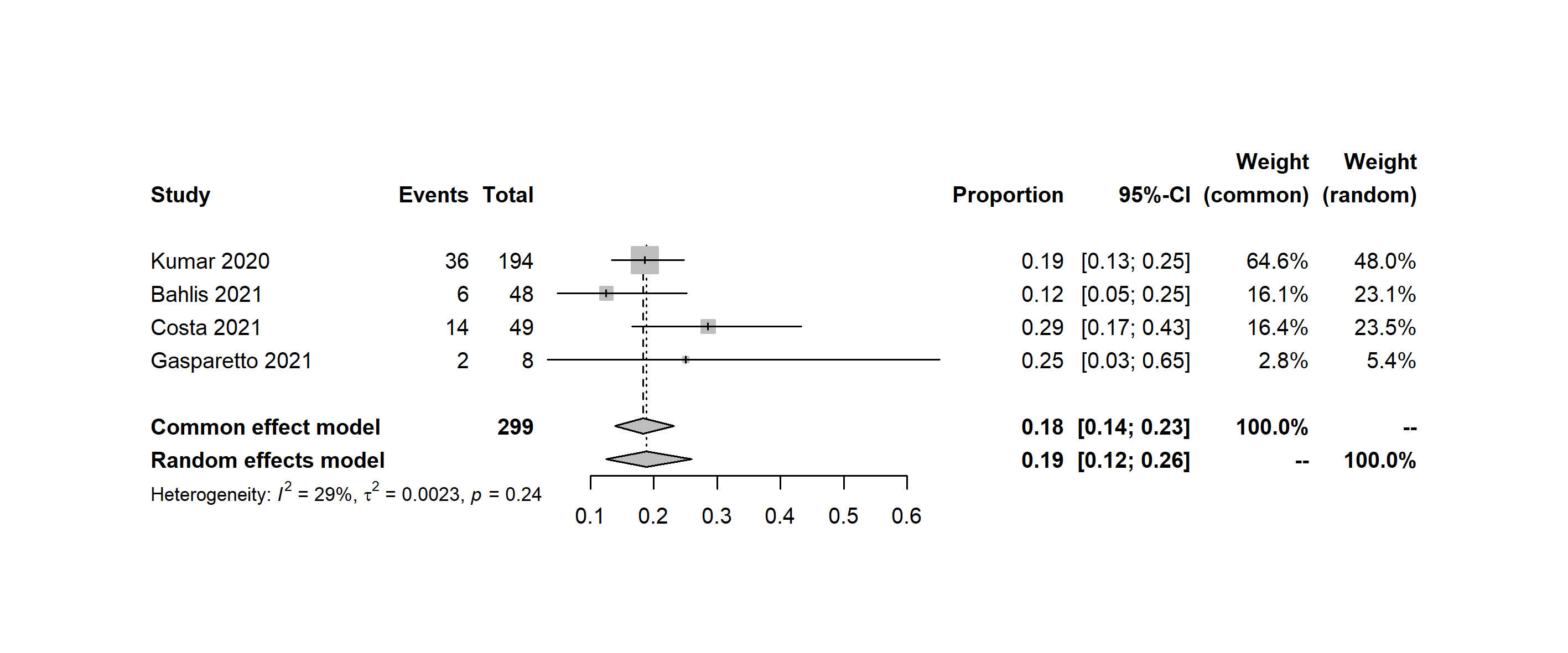
**Figure S8**. Forest plot of vomiting


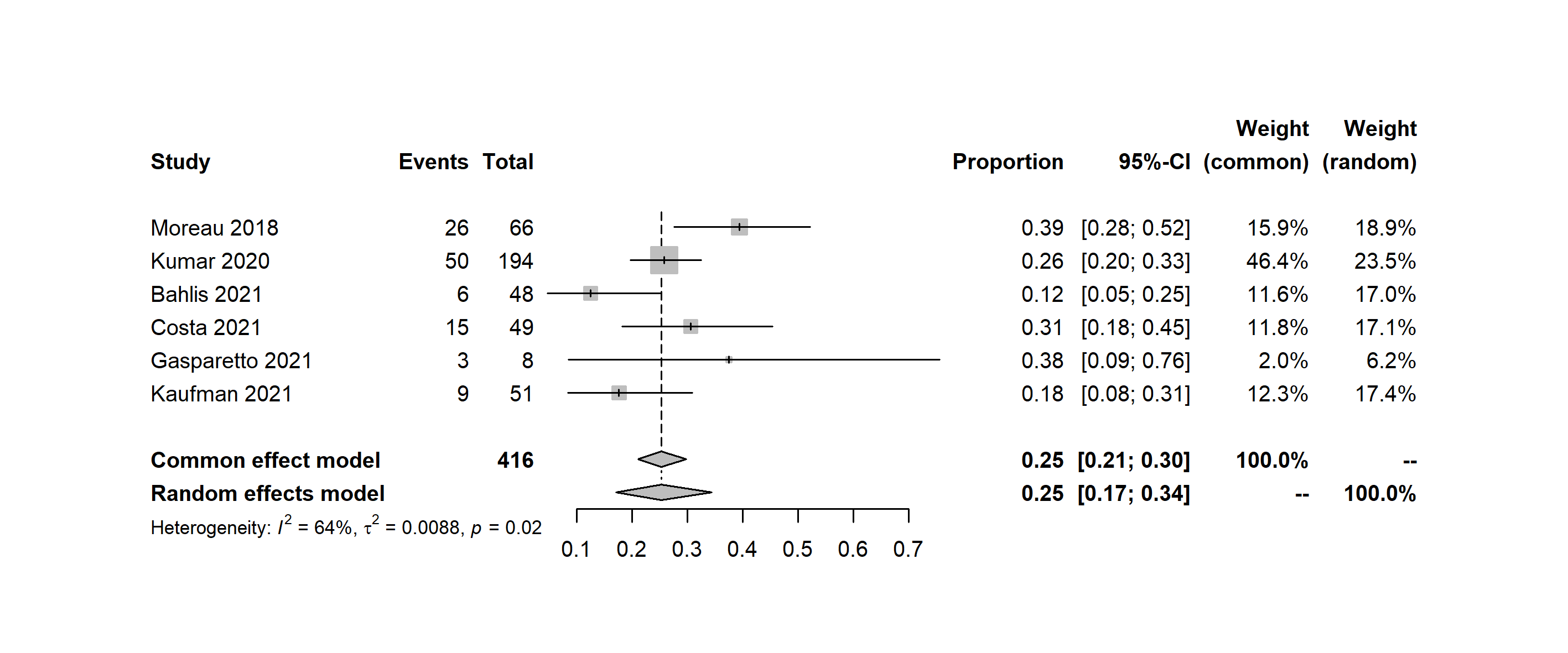
**Figure S9**. Forest plot of thrombocytopenia


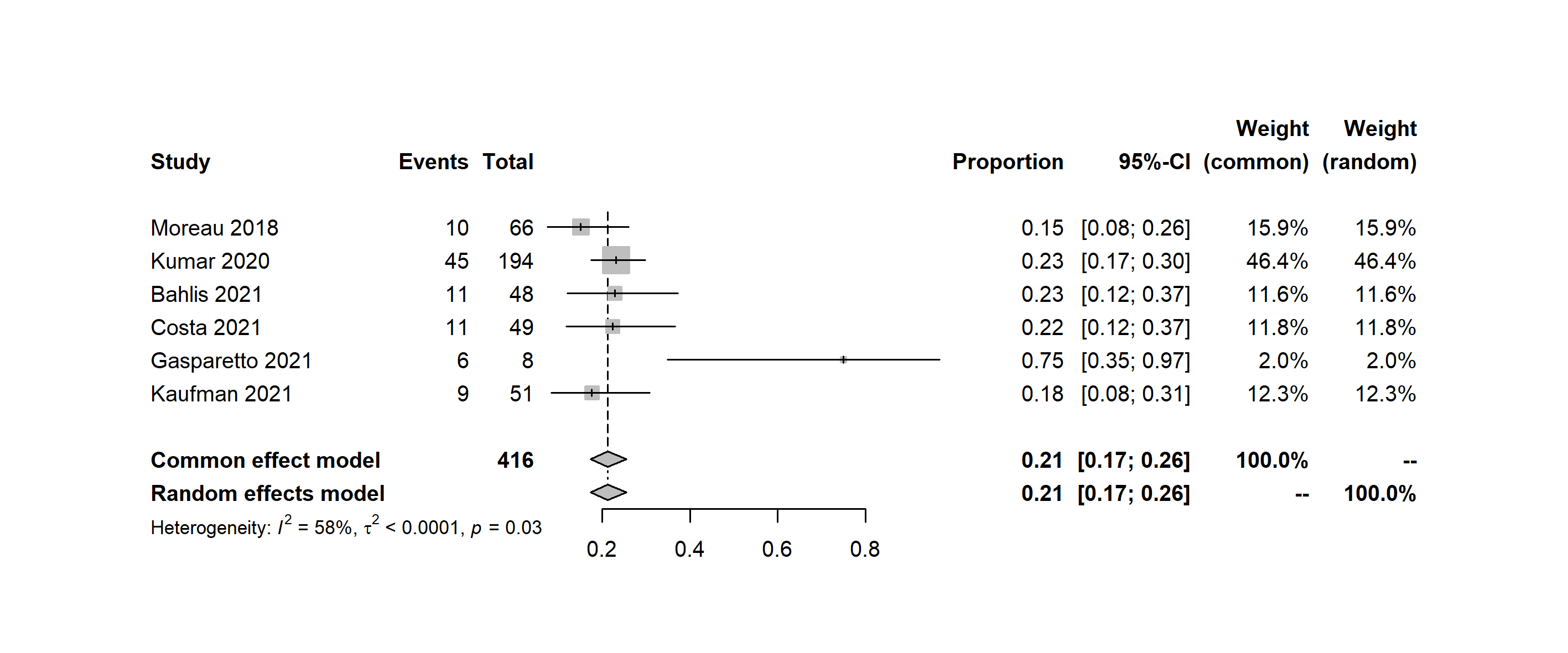
**Figure S10**. Forest plot of neutropenia


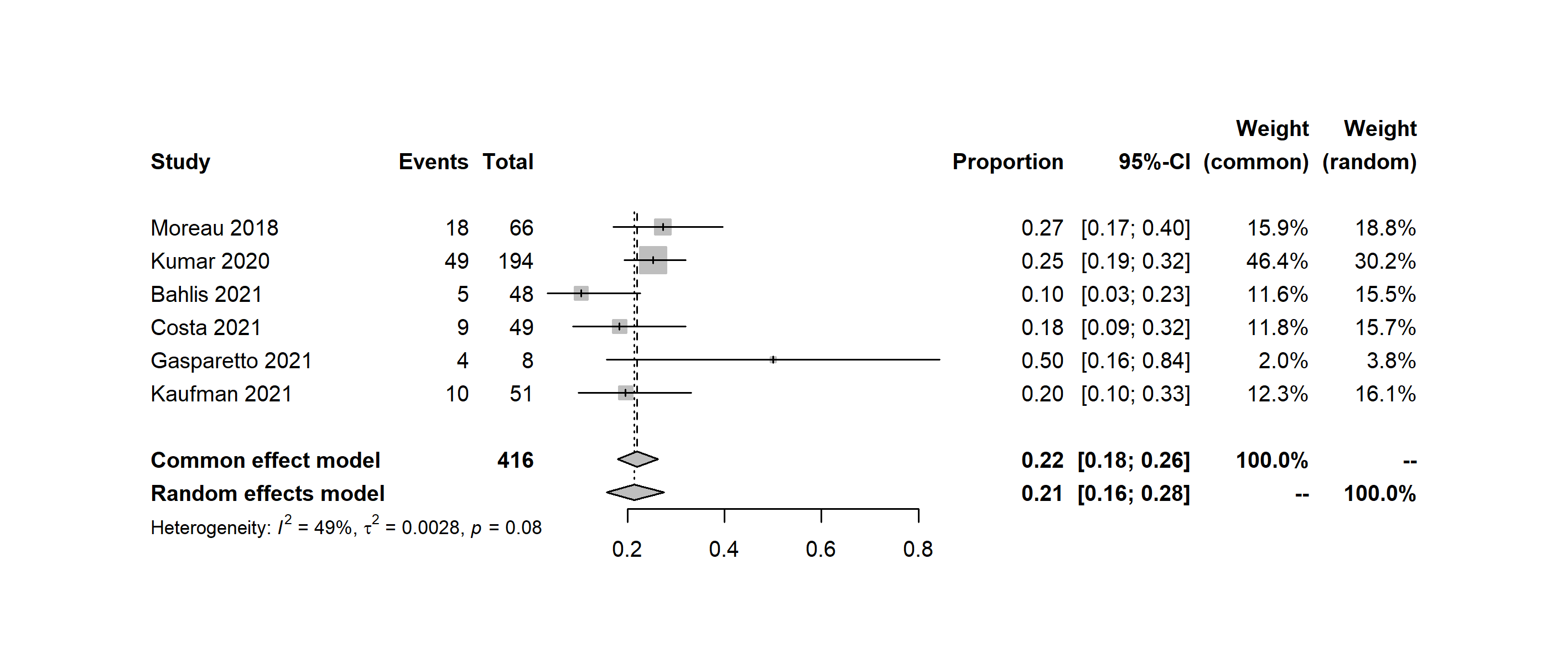
**Figure S11**. Forest plot of anemia


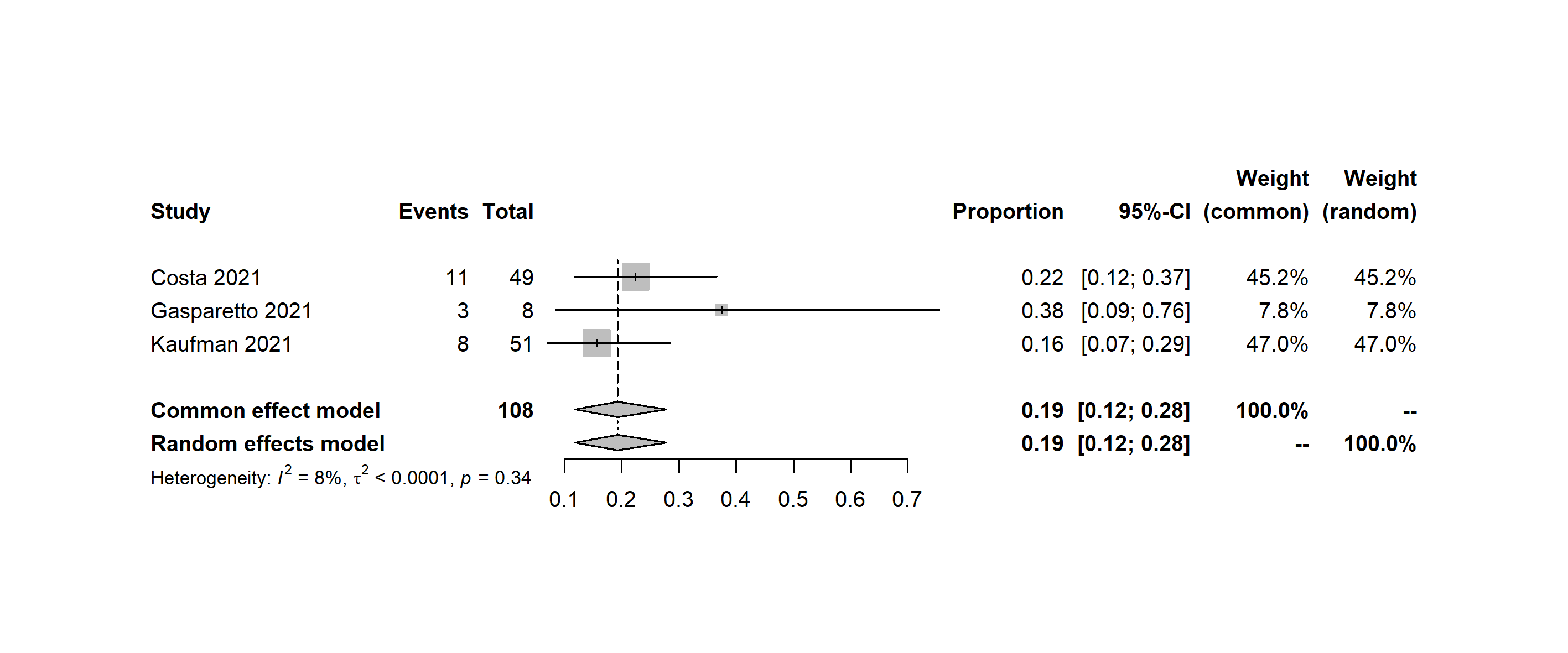
**Figure S12**. Forest plot of leukopenia


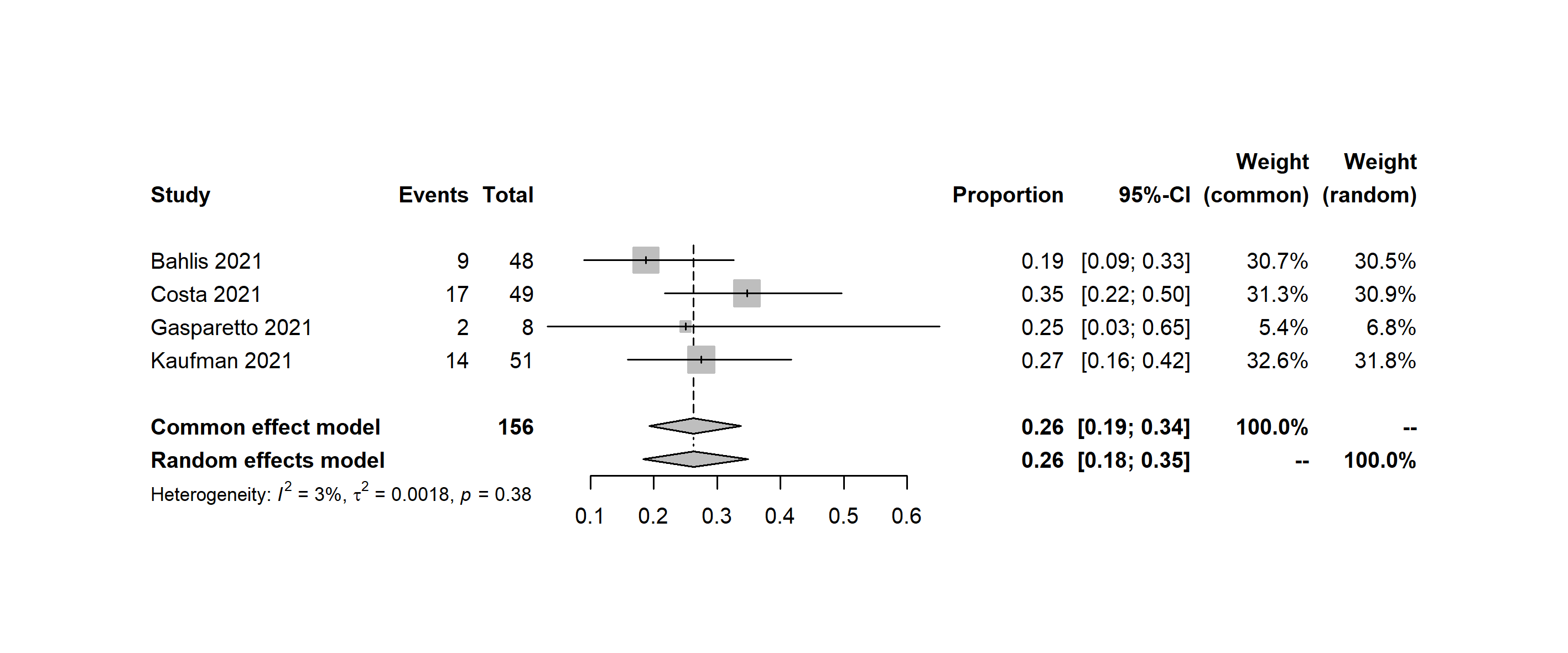
**Figure S13**. Forest plot of lymphopenia


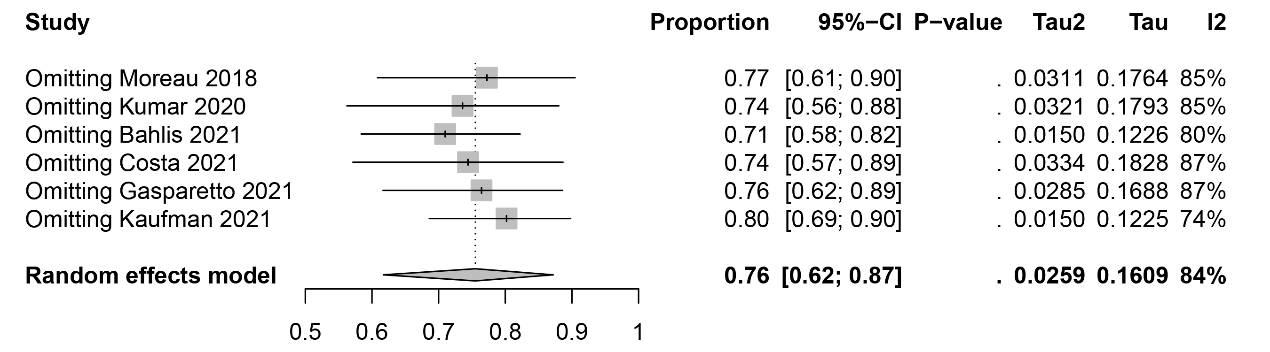


**Figure S14** Forest plot of sensitivity analysis on overall response rates


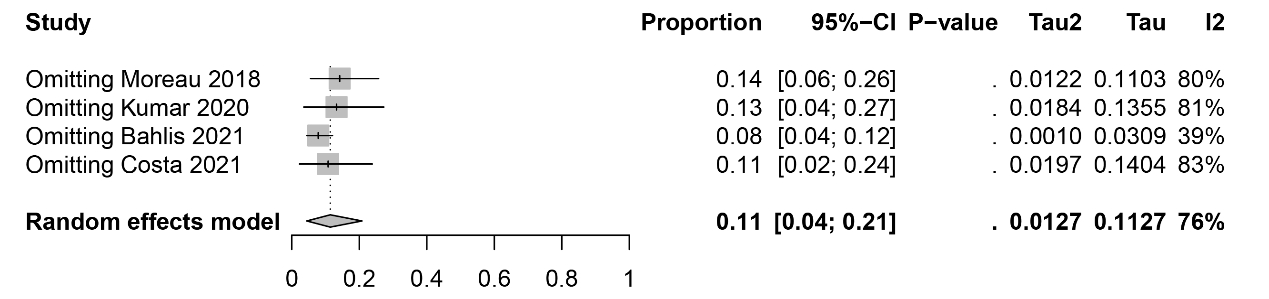


**Figure S15** Forest plot of sensitivity analysis on stringent complete response rates


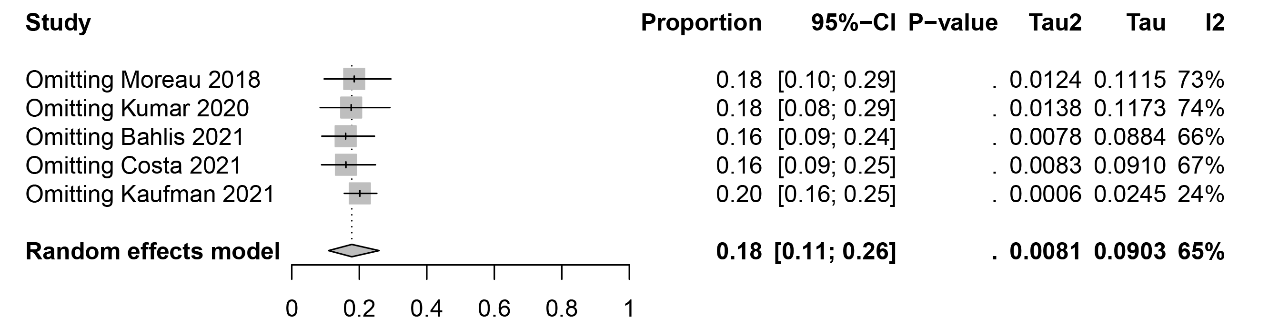


**Figure S16** Forest plot of sensitivity analysis on complete response rates


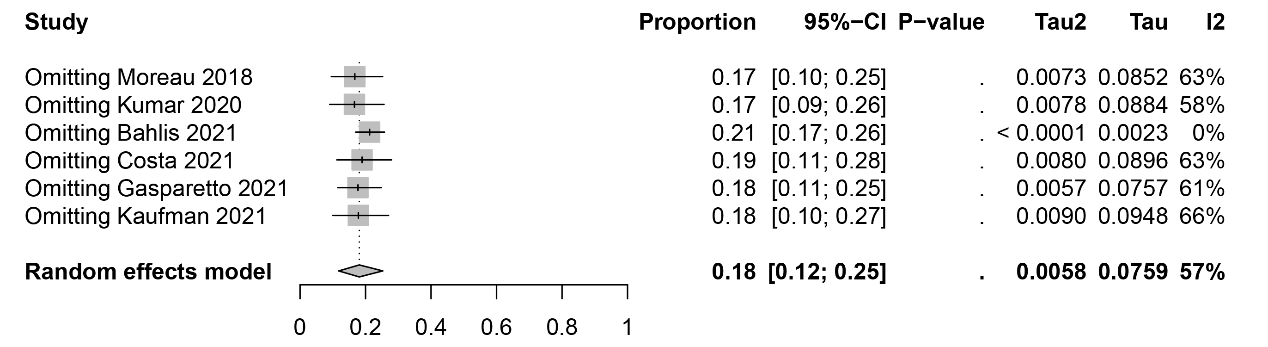


**Figure S17** Forest plot of sensitivity analysis on very good partial response rates


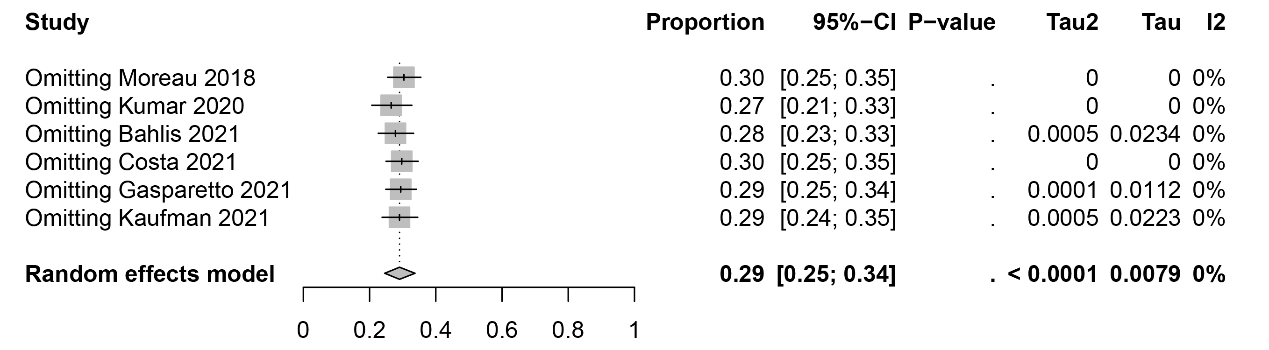


**Figure S18** Forest plot of sensitivity analysis on partial response rates


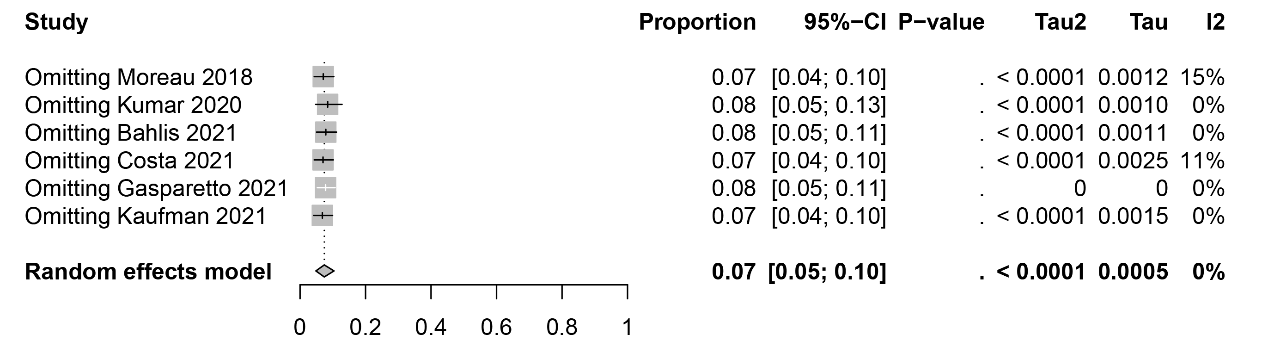


**Figure S19** Forest plot of sensitivity analysis on stable disease


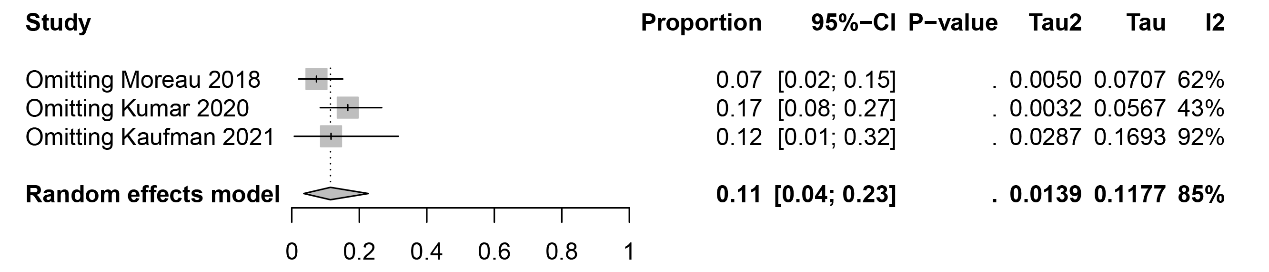


**Figure S20** Forest plot of sensitivity analysis on progressive disease


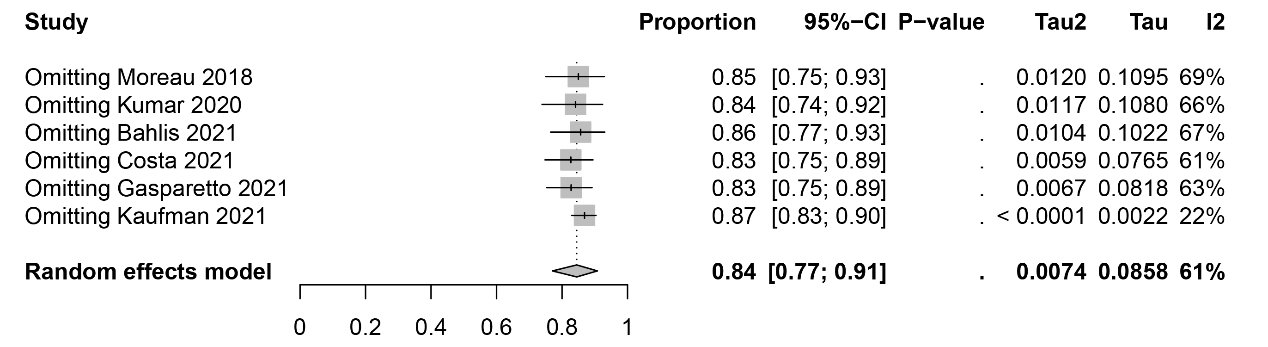


**Figure S21** Forest plot of sensitivity analysis on adverse events rates ≥ Grade 3

**Table S1** search history

**Date: 2022/11/07**

**PubMed**

| Search number | Query | Sort By | Filters | Search Details | Results | Time |
| --- | --- | --- | --- | --- | --- | --- |
| 5 | ((BCL-2[Title/Abstract]) OR (venetoclax[Title/Abstract])) AND (multiple myeloma[Title/Abstract]) | | Clinical Trial, English | (("BCL-2"[Title/Abstract] OR "venetoclax"[Title/Abstract]) AND "multiple myeloma"[Title/Abstract]) AND ((clinicaltrial[Filter]) AND (english[Filter])) | 19 | 9:08:47 |
| 4 | ((BCL-2[Title/Abstract]) OR (venetoclax[Title/Abstract])) AND (multiple myeloma[Title/Abstract]) | | English | (("BCL-2"[Title/Abstract] OR "venetoclax"[Title/Abstract]) AND "multiple myeloma"[Title/Abstract]) AND (english[Filter]) | 659 | 9:08:37 |
| 3 | ((BCL-2[Title/Abstract]) OR (venetoclax[Title/Abstract])) AND (multiple myeloma[Title/Abstract]) | | | ("BCL-2"[Title/Abstract] OR "venetoclax"[Title/Abstract]) AND "multiple myeloma"[Title/Abstract] | 715 | 9:08:22 |
| 2 | multiple myeloma[Title/Abstract] | | | "multiple myeloma"[Title/Abstract] | 43,945 | 9:08:08 |
| 1 | (BCL-2[Title/Abstract]) OR (venetoclax[Title/Abstract]) | | | "BCL-2"[Title/Abstract] OR "venetoclax"[Title/Abstract] | 69,147 | 9:07:26 |

**Embase**

| No. | Query | Results | Date |
| --- | --- | --- | --- |
| #5 | #4 AND 'clinical trial'/de | 146 | 7-Nov-22 |
| #4 | #1 AND #2 AND [english]/lim | 1276 | 7-Nov-22 |
| #3 | #1 AND #2 | 1361 | 7-Nov-22 |
| #2 | 'multiple myeloma':ab,ti | 76662 | 7-Nov-22 |
| #1 | venetoclax:ab,ti OR 'bcl 2':ab,ti | 87784 | 7-Nov-22 |

**Web of Science**

| **#** | **Search Query** | **Database** | **Results** | **Date Run** |
| --- | --- | --- | --- | --- |
| **1** | **venetoclax (Topic) OR BCL-2 (Topic)** | **Web of Science Core Collection** | **84660** | **Mon Nov 7 2022 22:20:39 GMT+0800 (中国标准时间)** |
| **2** | **multiple myeloma (Topic)** | **Web of Science Core Collection** | **72448** | **Mon Nov 7 2022 22:20:39 GMT+0800 (中国标准时间)** |
| **3** | **#1 AND #2** | **Web of Science Core Collection** | **1303** | **Mon Nov 7 2022 22:20:39 GMT+0800 (中国标准时间)** |
| **4** | **#1 AND #2 and English (Languages)** | **Web of Science Core Collection** | **1293** | **Mon Nov 7 2022 22:20:39 GMT+0800 (中国标准时间)** |
| **5** | **#1 AND #2 and English (Languages) and Article (Document Types)** | **Web of Science Core Collection** | **945** | **Mon Nov 7 2022 22:20:39 GMT+0800 (中国标准时间)** |

**Cochrane Library**

venetoclax in Title Abstract Keyword AND multiple myeloma in Title Abstract Keyword
